# Melatonin attenuates detrimental effects of diabetes on the niche of mouse spermatogonial stem cells by maintaining Leydig cells

**DOI:** 10.1038/s41419-018-0956-4

**Published:** 2018-09-20

**Authors:** Zhaoyu Du, Shuanshuan Xu, Shuxian Hu, Hong Yang, Zhe Zhou, Kuldip Sidhu, Yiliang Miao, Zhonghua Liu, Wei Shen, Russel J. Reiter, Jinlian Hua, Sha Peng

**Affiliations:** 1 College of Veterinary Medicine, Shaanxi Centre of Stem Cells Engineering & Technology, Northwest A&F University, Yangling, 712100 Shaanxi China; 2 Centre for Healthy Brain Ageing, UNSW Medicine, Randwick, NSW 2031 Australia; 3 College of Animal Science & Technology, College of Veterinary Medicine, Huazhong Agricultural University, 430070 Wuhan, China; 4 College of Life Science, Northeast Agricultural University, 150036 Harbin, China; 5 College of life sciences, Institute of Reproductive Sciences, Qingdao Agriculture University, 266109 Qingdao, China; 6 Department of Cell Systems and Anatomy, UT Health, San Antonio, TX78229-3900 USA

## Abstract

Diabetes mellitus affects a large number of men of reproductive age and it usually leads to serious reproductive disorders. However, the underlying mechanisms and specific therapies still remain largely unknown. We observed Leydig cell loss in the testes of diabetic mice. Continuous high glycemic status of testes stimulated expression of Caspase12, Grp78, and Chop, the three ERS response factors; this might induce cell cycle arrest and apoptosis of Leydig cells in response to ERS. In these diabetic mouse models, melatonin alleviated apoptosis of testicular stromal cell induced by ERS, and promoted SSCs self-renewal by recovering Leydig cells secretion of CSF1 after 8 weeks of treatment. To explore the relationship between CSF-1 and ERS in Leydig cells, we treated Leydig tumor cell line with an activator Tuniamycin and an inhibitor 4-Phenylbutyrate of ERS. Our data showed that the CSF-1 expression in mouse Leydig cell lines decreased six-fold while reversely increasing five-fold in the 4-Phenylbutyrate-treated group. Thus, melatonin likely alleviates the loss of Leydig cells in diabetic testes and provides a healthier niche for SSCs to self-renew and continually provide healthy sperm for male fertility.

## Introduction

Diabetes mellitus (DM) is a major cause of large-scale morbidity and mortality^1^. It is a syndrome that adversely affects all physiological systems^2^ including the deleterious effects on the male reproductive system both in diabetic men and male animals^3,4^.

Male fertility relies on the continuity of spermatogenesis in the testes^5^ and SSCs that undergo self-renewal and differentiation compose the “fountainhead” of spermatogenesis^6^. SSCs are the sole germline stem cells, which sustain self-renewal and division to replenish the population and generate progenitor spermatogonia for differentiation^7^. The fate of SSCs are influenced by a niche microenvironment composed of a growth factor milieu provided by several testicular somatic-supporting cell populations^5^. In mammalian testes, Sertoli cells, which are the major contributors to the SSC niche^8,9^, play a pivotal role in spermatogenesis. Previous study has indicated that Sertoli cell metabolism is influenced by a testosterone deficiency in progressive stages of DM^10^ and by the glucose homeostasis which is controlled by the combined action of insulin and melatonin^11^. Disturbance of these regulatory factors may explain male infertility induced due to diabetes since spermatogenesis is supported by Sertoli cell growth factors and transcription factors^12^. A disturbance of testosterone synthesis by Leydig cells in testicular interstitial tissue are also disordered in diabetic testis^13^. In the fetal mouse testis, both Sertoli and Leydig cells are required for testosterone synthesis, while the adult Leydig cells synthesize testosterone to maintain male reproductive function^14^. Thus Sertoli and Leydig cells both play crucial roles in the establishment of the niche microenvironment for SSCs. In addition, interstitial Leydig cells express CSF1, which also stimulates the self-renewal of SSCs in mice^15^. Although the impact of diabetes on Sertoli cell metabolism and testosterone synthesis is becoming increasingly clear, its effect on SSCs self-renewal and differentiation supported by Leydig cells are not well known.

ERS occurs when the ER function becomes perturbed by hypoxia and hypoglycemia, and protein misfolding during biosynthesis^16^. Modulation of ERS maintains the balance between survival and death by regulating autophagy and apoptosis under different stressful conditions. ERS is involved in diabetes-induced testicular cell death^17,18^ and spermatogenesis impairment by reducing testosterone production by Leydig cells^19^. Leydig cells, also known as interstitial cells of Leydig, are found adjacent to the seminiferous tubules in the testicle. Leydig cells produce testosterone in the presence of luteinizing hormone (LH). As Leydig cell is an important part of the male reproductive microenvironment, ERS in diabetic testis could be a major factor to the damage of Leydig cells and inhibit the Leydig cells from supporting the spermatogenesis.

Melatonin, is an indole synthesized and secreted by the pineal gland; its concentrations in the blood vary daily and seasonally in mammals^20,21^. Melatonin prevents various ERS-related diseases and restores the cells damage caused by ERS^22,23^. Melatonin also plays a significant role in the regulation of self-renewal and differentiation of various stem cells, including mesenchymal stem cells^24^ and spermatogenic cells^25^. Moreover, melatonin prevents testicular damage caused by environmental toxins and drugs^26–28^ based on its characteristics of lipophilic and hydrophilic free radical scavengers^29,30^. Whether melatonin prevents ERS in the Leydig cells and then protects the self-renewal capacity of SSCs under high glucose conditions is still unknown.

This study is designed to establish hyperglycemia as a major physiological determinant in SSC microenvironment and demonstrates the direct relationship of the regulation of high-glucose-induced ERS with the specification and maintenance of SSCs. We tested the hypothesis as to whether melatonin application is sufficient to rescue diabetic male fertility damage via inhibition of ERS and activation of SSC self-renewal.

## Results

### Diabetes caused Leydig cell loss in mouse testes

**Fig. 1:**
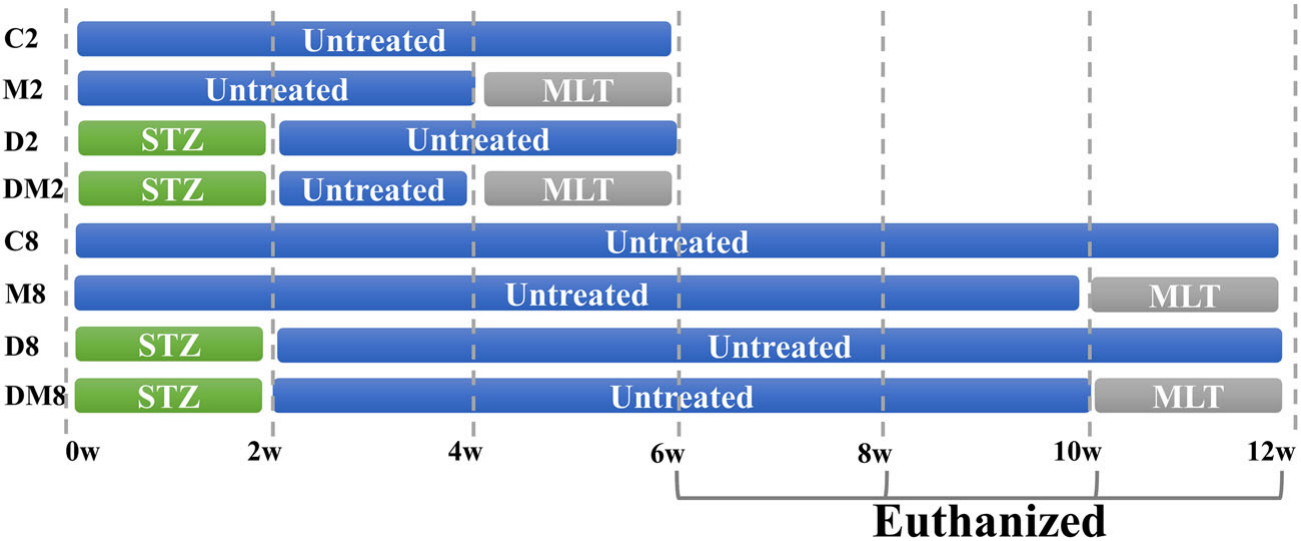
The schematic of experimental diabetic mouse models prepared using STZ. Melatonin (MLT) was given by gavage daily (10 mg/kg/d) for 2 or 8 weeks. Diabetes was induced by a single dose of streptozotocin (STZ) by intra-peritoneal injection (100 mg/kg) on the first day. C2 and C8: two-week and eight-week controls, M2 and M8: two-week and eight-week groups treated with melatonin daily during the period, D2 and D8: two-week and eight-week diabetic, DM2 and DM8: two-week and eight-week diabetic treated with melatonin. Euthanasia was performed at the 6th and 12th week. MLT: melatonin

**Fig. 2:**
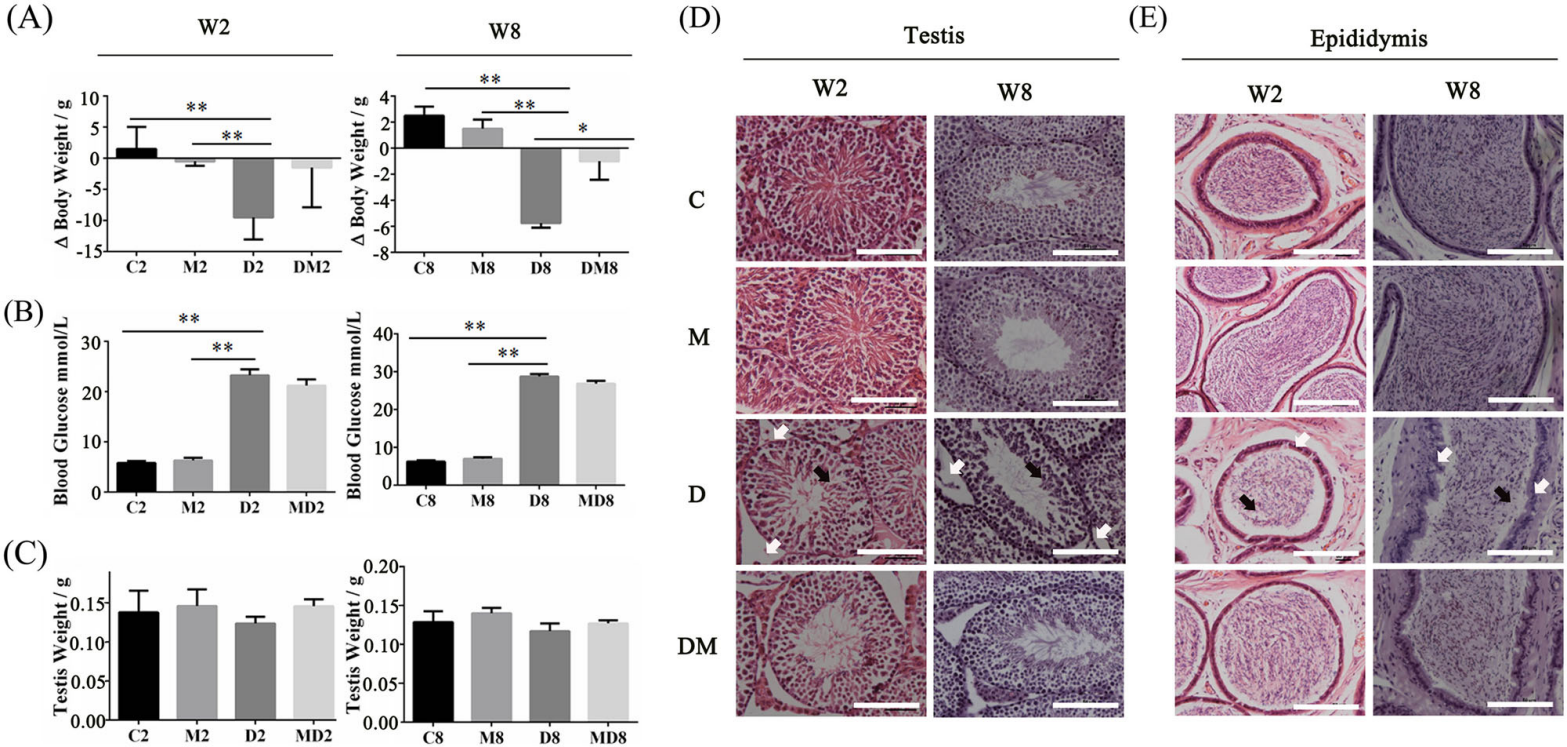
Melatonin reduced testicular and epididymal injuries caused by diabetes. **a** The body weight gain in the four different treatment groups in the short term (W2, 2 weeks) and long term (W8, 8 weeks) experiments. **b** Blood glucose testing in the four different groups in W2 and W8 experiments. **c** Testis weight measured in the four different groups in W2 and W8 experiments. **d** Testicular hematoxylin-eosin (HE) histopathological sections of four different treatment groups in W2 and W8 experiments. **e** Epididymal HE hispathology sections of four different treatment groups in W2 and W8 experiments. Scale bar, 100 μm. (The results are expressed as the mean ± S.E.M of at least three mice (*n* = 3) and the statistical significance is expressed as follows: *p < 0.05; **p < 0.01). C2 and C8: two-week and eight-week control, M2 and M8: two-week and eight-week groups treated with melatonin daily during the period, D2 and D8: two-week and eight-week diabetic, DM2 and DM8: two-week and eight-week diabetic treated with melatonin

Figure 1 describes the schematic for the experimental plan using ICR mice that were treated with STZ and melatonin. The body weight gain (Fig. 2a) did not vary between D2 and DM2 in the short term experimental groups. Both diabetic groups, particularly the melatonin treatment group, from the longer-duration experiment exhibited a slower weight loss. Blood glucose control was not significantly improved by melatonin (Fig. 2b). The testicular weight was not affected by melatonin treatment under healthy and diabetic conditions in short- and long-term experiments (Fig. 2c). In Figures S1A and S1B, 8-week diabetes induced mild puffiness and congestion of the testicles. Conversely, this condition was relieved by melatonin. To examine the cause of the damaged tissues, the morphology of the testes was examined at the light microscopic levels. The findings indicated that hyperglycemia resulted in the loss of Leydig cells (white arrows) and SSCs (black arrows) and promoted the thickening of the basement membrane in the seminiferous tubules (Fig. 2d) in short term and long term experiments. The sperm density in the epididymis of the diabetic mice was significantly reduced (black arrows), and the integrity of the epididymal duct wall was disrupted (white arrows) when melatonin treatment was not administered (Fig. 2e).

### Melatonin improved cell survival in the testis with hyperglycemia by decreasing ERS

**Fig. 3:**
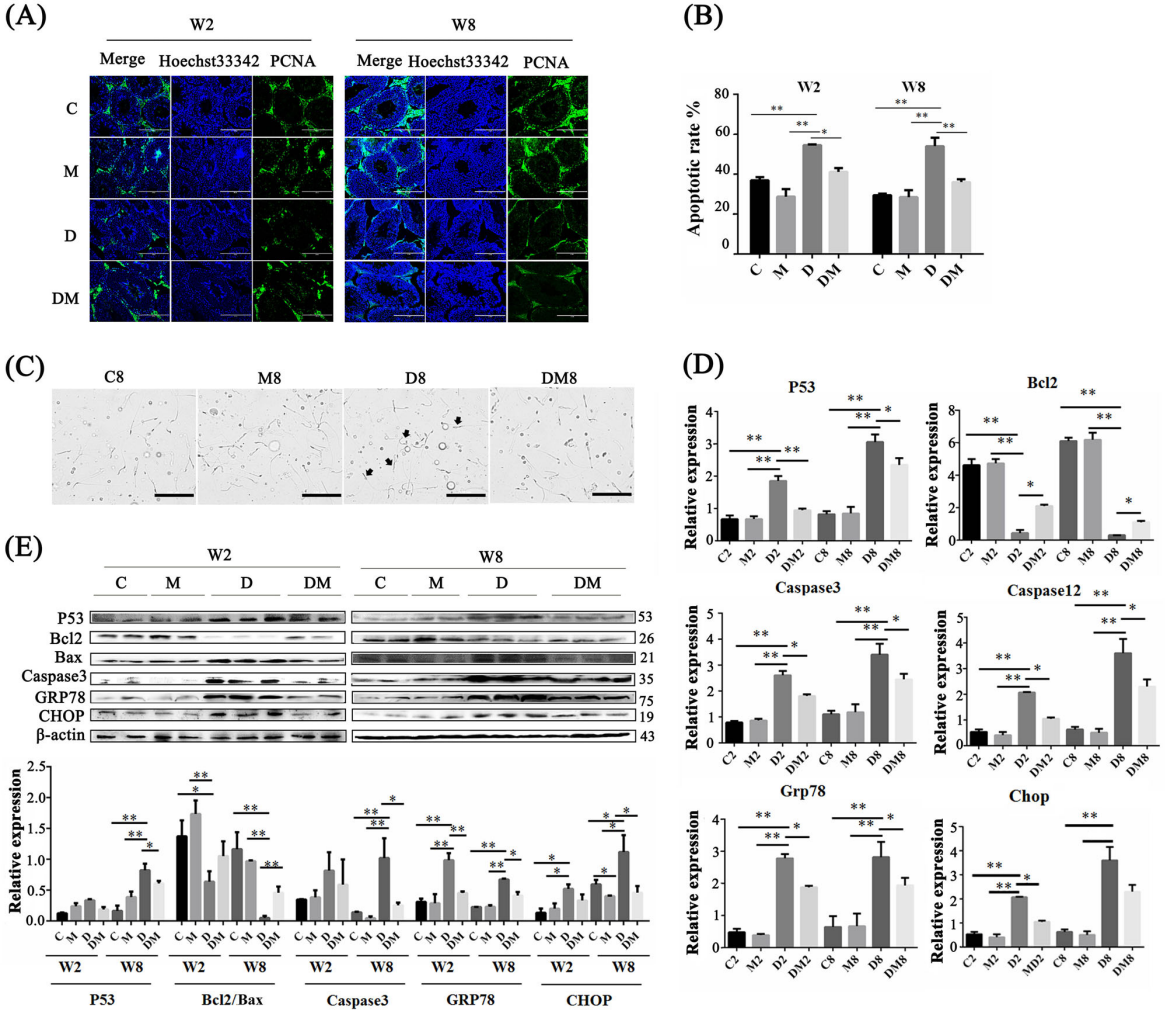
Effect of melatonin on the proliferation, cell survival, and spermatogenic function of testicular tissue. **a** PCNA expression in testes of four different treatment groups in short term (W2, 2 weeks) and long term (W8, 8 weeks) experiments. Scale bar, 200 μm. **b** Cell apoptosis rate was determined by flow cytometry (FCM). **c** Epididymal semen smears of four different groups in W8 experiments. Scale bar, 100 μm. Black arrows in D8 show the abnormal characteristics. **d** RT-qPCR analysis of apoptosis-related genes (*P53, Bcl2, Caspase 3* and *Caspase 12*) and endoplasmic reticulum stress-related genes (Grp78 and Chop) in testicular cells suspensions of four different groups in W2 and W8 experiments. **e** Western blot analysis of apoptosis-related genes in testicular cell suspensions of four different groups in W2 and W8 experiments. The histograms show quantitative results of Image J (V1.48d) gradation analysis for the western blot experiments. (The results are expressed as the mean ± S.E.M of at least three mice (*n* = 3) and the statistical significance is expressed as follows: *p < 0.05; **p < 0.01). C2 and C8: two-week and eight-week control; M2 and M8: two-week and eight-week groups treated with melatonin (MLT) daily during the period; D2 and D8: two-week and eight-week diabetic; DM2 and DM8: two-week and eight-week diabetic treated with MLT

To explore the causes of cell loss, the expression of PCNA was examined; this is a specific markers of cell proliferation. PCNA protein level was significantly lower (*P* < 0.05) in the diabetes group than in the control group (Fig. 3a). The proportion of PCNA-positive cells in the DM group increased in short- and long-term experiments. Anti-apoptosis is regarded as an essential function of melatonin in reproductive cells^31^. Melatonin could substantially reduce the number of apoptotic cells (Fig. 3b and S2A) from 54.4% to 39.8% in the 2-week group and from 51.8% to 37.9% in the 8-week group. Different ploidy types of cells were counted by flow cytometry because haploid testicular cells, especially spermatids, were included (Fig. S2B). In short- and long term experiments, the low proportion of the tetraploid cells in the testes of the diabetic mice reached 10.5% and 12.1%, respectively. This observation implied that cell proliferation declined. The proportion of the haploid cells in 8-week diabetics decreased from 56.7% to 51.6%, which might be the direct cause of reduced fertility; this proportion recovered to 54.1% in the DM group (Fig. S2B). In further experiments, sperm with abnormal characteristics, such as unusually large heads, sickle-shaped heads, and curly tails, were found in semen on the epididymis smear obtained from the diabetic mice (Fig. 3c). Moreover, melatonin therapy protected the sperm from damage by diabetes (Fig. 3C).

To investigate how melatonin decreases cell apoptosis in the testes, the gene expression profile was analyzed in the testicular tissue from the mice with and without melatonin therapy. RT-qPCR analysis demonstrated that the expression levels of *p53* and *caspase3* in melatonin-treated diabetic mice were significantly lower (*P* < 0.01). By comparison, the expression level of *Bcl2* was higher than that in diabetic mice (Fig. 3d). In Fig. 3e, the Bcl2/Bax ratio was significantly (*P* < 0.01) enhanced and the *p53* and *caspase3* expression levels decreased in melatonin-treated diabetic testes. Cell loss induced by excessive apoptosis occurred in the diabetic mice. Apoptosis is associated with many chronic inflammatory diseases, including diabetes, and ERS is one of the main causes of apoptosis. After a 2-week melatonin treatment, the mRNA expression levels of the detected ERS markers, including *Caspase12*, *Grp78*, and *Chop*, were lower than those in the diabetic mice (Fig. 3d). In Fig. 3f, g, the protein expression levels of CHOP and Grp78 were reduced. These data imply that hyperglycemia caused an imbalance of homeostasis in the ER and thus stimulated ERS. ERS exacerbation might aggravate testicular injury in diabetic animal models.

### Melatonin protected Leydig cells under high glucose condition

**Fig. 4:**
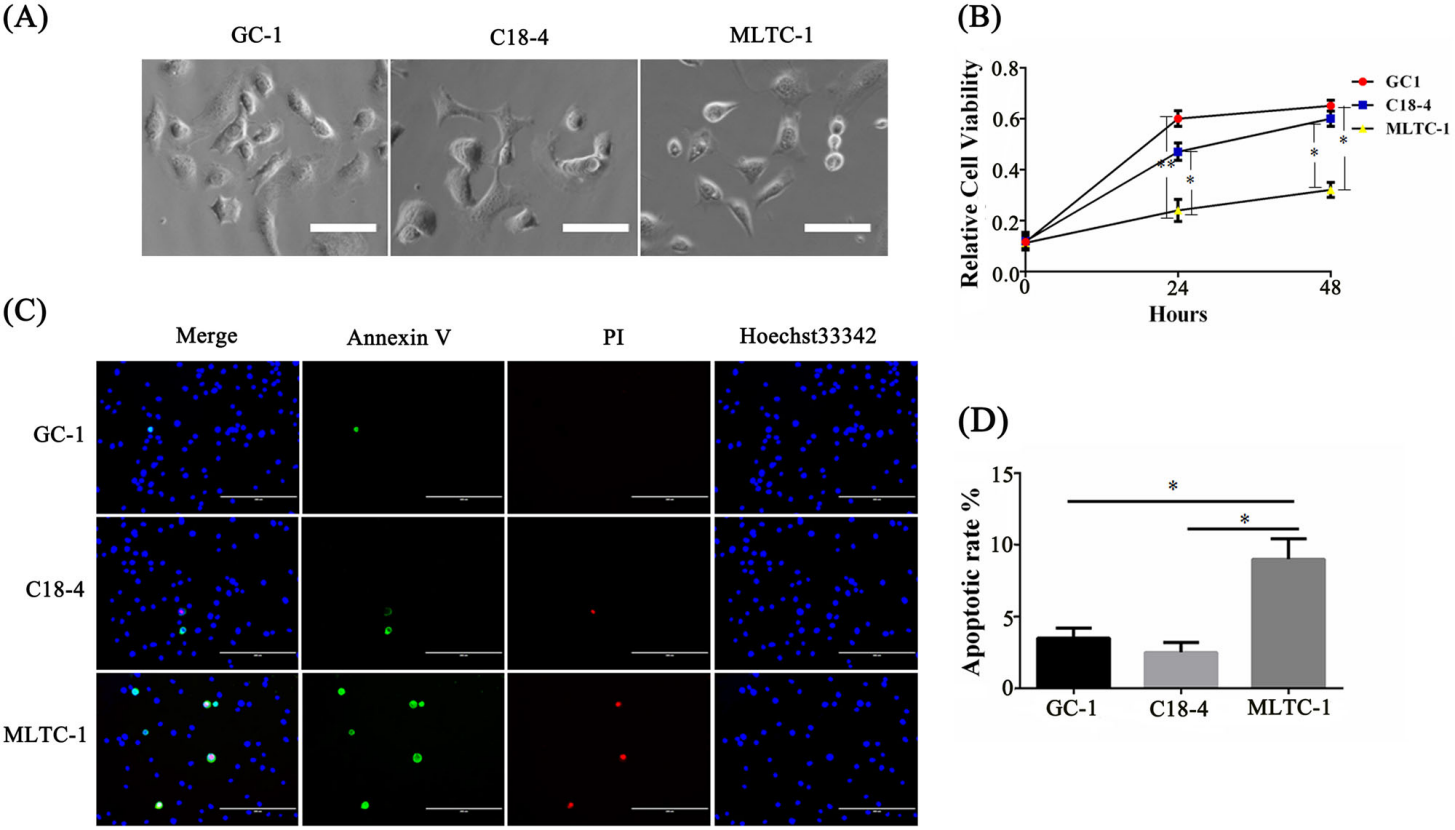
Leydig cells showed a higher sensitivity to high concentrations of glucose compared to SSCs. Two different mouse spermatogonial stem cell lines (GC-1 and C18-4) and one mouse Leydig cell line (MLTC-1) were treated with high concentrations of glucose (20 mM). **a** Morphological analysis of the three cell lines treated with high concentration of glucose for 24 h. Scale bar, 100 μm. **b** CCK8 assay detected the relative cell viability of the three cell lines under high concentrations of glucose at three time points (0 h, 24 h, 48 h). **c** Annexin V-FITC/PI assay assessed the cell apoptosis rate of the cell lines after 24 h treatment with high concentration of glucose. Scale bar, 200 μm. **d** Histogram was the statistical apoptosis rate for **c**. (The results are shown as the mean ± S.E.M of five separated wells of cells (*n* = 5) in at least three different experiments and the statistical significance is expressed as follows: *p < 0.05; **p < 0.01.)

In Figs. 2d and 3a, Leydig cells experienced the most severe loss and the most evident proliferative inhibition in diabetic mouse testes. First, the glucose sensitivity of different types of testicular cells was compared. Two mouse SSC lines (GC-1 and C18-4) and one Leydig tumor cell line (MLTC-1) were treated with 20 mM glucose. After 24 h of incubation with high glucose, the morphological characteristics of the MLTC-1 cells were significantly changed; that is, they shrank and became round (Fig. 4a). The viability of the MLTC-1 cell decreased to a greater extent than that of two other SSC lines (Fig. 4b). The proportion of apoptotic MLTC-1 cells reached 9% (Fig. 4c, d), which was significantly higher than that of the two other groups.

**Fig. 5:**
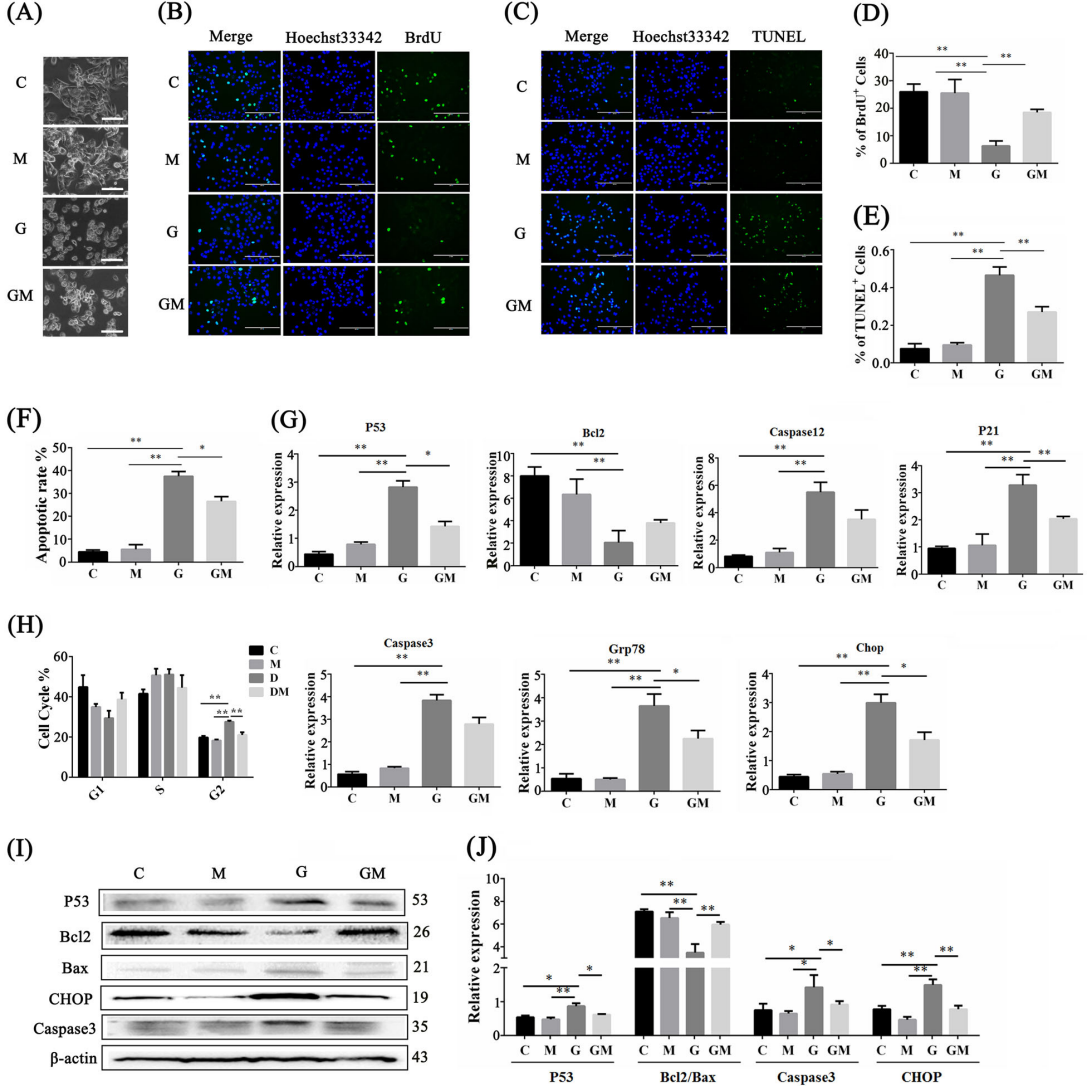
Melatonin accelerated the proliferation of Leydig cells and restrained its apoptosis. **a** Morphological comparison of MLTC-1 was performed when cultured for 48 h in four different groups (C control, M melatonin treatment, G high glucose treatment, GM high glucose in combination with melatonin treatment). Scale bar, 100 μm. **b**–**d** BrdU incorporation assay detected the proliferative ability in four different groups which were all treated for 48 h. Immunofluorescence images show an increase in incidence of BrdU-positive cells in GM group compared to G group. Scale bar, 200 μm. Histogram was the statistical BrdU-positive rate for **b**. **c**–**e** TUNEL assay detected the apoptosis rate in four different groups, which were all treated for 48 h. Immunofluorescence images show a decrease in incidence of TUNEL-positive cells in GM group compared to G group. Scale bar, 200 μm. Histogram is the statistical TUNEL positive rate for **c**. **f**–**h** MLTC-1 apoptosis rate (**f**) and cell cycle distribution (**h**) was determined by FCM in four different treatments. **g** RT-qPCR examined the expression of apoptosis-related genes (*P53, Bcl2, Caspase3, Caspase12* and *P21*) and ERS-related genes (*Grp78* and *Chop*) in the four different groups of MLTC-1. **i** Western blot analysis of apoptosis- and ERS-related genes in MLTC-1 under four different treatments. **j** Histograms show quantitative results of Image J (V1.48d) gradation analysis for the western blot experiments in **i**. (The results are shown as the mean ± S.E.M of four separated wells of cells (*n* = 4) in at least three different experiments and the statistical significance is expressed as follows: *p < 0.05; **p < 0.01.)

MLTC-1 cells were treated with 20 mM glucose and 1 μM Melatonin. After 24 h, significant differences were observed. In particular, compared with the cells in the control group (C) and the melatonin group (M), the cells in the high-glucose group (G) shrank and became round, exhibited a loose arrangement in a monolayer, lost adhesion properties, and floated on the medium. The viability of the cells under high glucose in combination with melatonin treatment (GM) was recovered (Fig. 5a). BrdU positive incidence of MLTC-1 recovered from 7.6% in G groups to 19% in GM groups (Fig. 5b–d). Meanwhile, the excessive apoptosis decreased from 43% in G groups to 23% in GM groups (Fig. 5c–e). The apoptosis assay also confirmed that melatonin lowered the high apoptotic rate caused by high glucose (Fig. 5f and S3A). To investigate how high glucose inhibited MLTC-1 proliferation, the cell cycle was analyzed by means of flow cytometry. In Fig. 5h and S3B, high glucose treatment contributed to the G2/M phase arrest of MLTC-1, which was relieved by melatonin treatment.

Diabetes increased ERS in whole testicular tissues, and MLTC-1 cells were highly sensitive and apoptotic under the high glucose condition, while the SSCs cell lines were less sensitive to high glucose. These findings suggested that high glucose induced excessive ERS in Leydig cells, which was alleviated by MLT. RT-qPCR and Western blot results indicated that ERS-related genes, including Grp78, Chop, and Caspase12, were increased under high-glucose condition in the MLTC-1 cells. High glucose-induced ERS triggered cell apoptosis, as marked by *P53*, *Bcl2*, *caspase3* and *P21* (Fig. 5g). Consistently, the protein expression levels of P53, Caspase3 and CHOP were elevated, whereas the protein expression level of Bcl2/Bax dropped (Fig. 5i, j).

### ERS in Leydig cells inhibited SSC self-renewal capacity

**Fig. 6:**
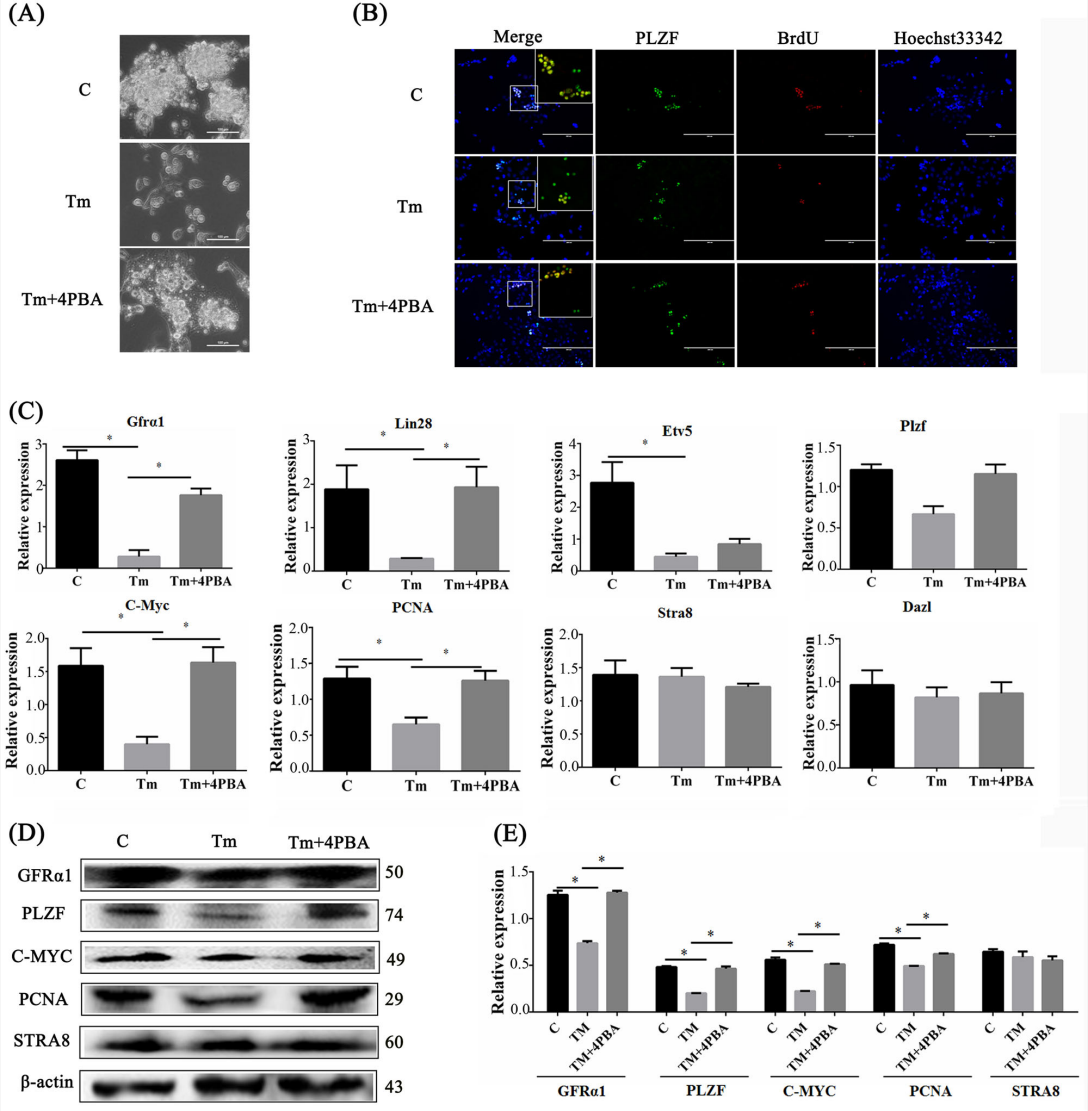
ERS states of Leydig cells affected the fate of SSCs. **a** Morphological comparison between mouse SSCs growing on the feeder of three different ERS states (C control, Tm Tm treatment induced excessive ERS, Tm + 4PBA Tm treatment in combination with 4PBA for alleviated ERS). Scale bar, 100 μm. **b** PLZF expression and BrdU assay fluorescence with Hoechst33342 and their merges in SSCs under the three different treatments. Scale bar, 200 μm. **c** RT-qPCR detected the self-renewal genes (*Gfrα1*, *Lin28*, *Etv5* and *Plzf*), proliferation-related genes (*C-Myc* and *PCNA*) and meiosis induced genes (*Stra8* and *Dazl*) in SSCs under the three different treatments. **d** Western blot analysis of self-renewal genes, proliferation-related genes and meiosis inducers in SSCs under the three treatments. **e** Histograms show the statistical result of Image J (V1.48d) gradation analysis for the western blot experiments in **d**. (The results are expressed as the mean ± S.E.M of three separated wells of cells (*n* = 3) in at least three different experiments and the statistical significance is expressed as follows: *p < 0.05; **p < 0.01)

On the basis of the findings, it was hypothesized that the increased ERS levels supported by a high-glucose environment promoted apoptosis of Leydig cells and contributed to the progression of male reproductive injury. To intervene in ERS caused by a high-glucose environment in Leydig cells, the MLTC-1 cells are treated with Tm and 4PBA, which, respectively, caused and inhibited excessive ERS. It was confirmed that high glucose and Tm triggered excessive ERS, and the cell activity remarkably decreased in the Tm-treated group; and this was effectively mitigated by melatonin and 4PBA (Figs S4A and S4B). Mitomycin C was then used to treat MLTC-1 cells pretreated by 4PBA in combination with Tm to produce the feeder layers for SSCs. When morphologically growing on the feeder pretreated with Tm, SSCs were unable to proliferate to form clones, while SSCs growing on the feeder layer treated with melatonin and 4PBA showed a significantly improved colony-formation ability (Fig. 6a). To determine the ability of SSC proliferation, SSCs were marked with PLZF antibody and a BrdU incorporation assay was performed. The results suggested that the pretreated feeder layer with Tm decreased the positive rate of BrdU SSCs (Fig. 6b). In the cells pretreated with Tm and 4PBA, the rate of the BrdU-labeled cells measured was found higher than that in the Tm-treated groups. These SSCs then grew rapidly into colonies as in the control group. Then, SSCs from different feeder layers were harvested and the markers of self-renewal and differentiation were estimated. In Fig. 6c, the mRNA expression levels of *Gfrα1*, *Lin28a*, *Etv5*, *Plzf, C-Myc*, and *PCNA* were sharply reduced in the SSCs growing on the feeders treated with Tm. In contrast, the expression levels of these genes increased when the cells grew on the feeder layers treated with Tm and 4PBA. Consistent with the mRNA expression pattern, the GFRα1, PLZF, C-MYC and PCNA protein expression levels were downregulated in the SSCs growing on the feeder layers under ERS (Fig. 6d, e). However, the expression levels of meiosis inducers, such as STRA8 and DAZL, did not significantly change (Fig. 6c–e).

### Melatonin protected CSF1 function against high glucose suppression in testis

Micro-environmental conditions called a niche are essential to SSC self-renewal and spermatogenesis. Considering the damaged Leydig cells, secretory functions of Leydig cells, and the important roles of CSF1 in SSC self-renewal, it was assumed that CSF1 played the most important role in melatonin maintenance of SSC self-renewal under high glucose conditions.

**Fig. 7:**
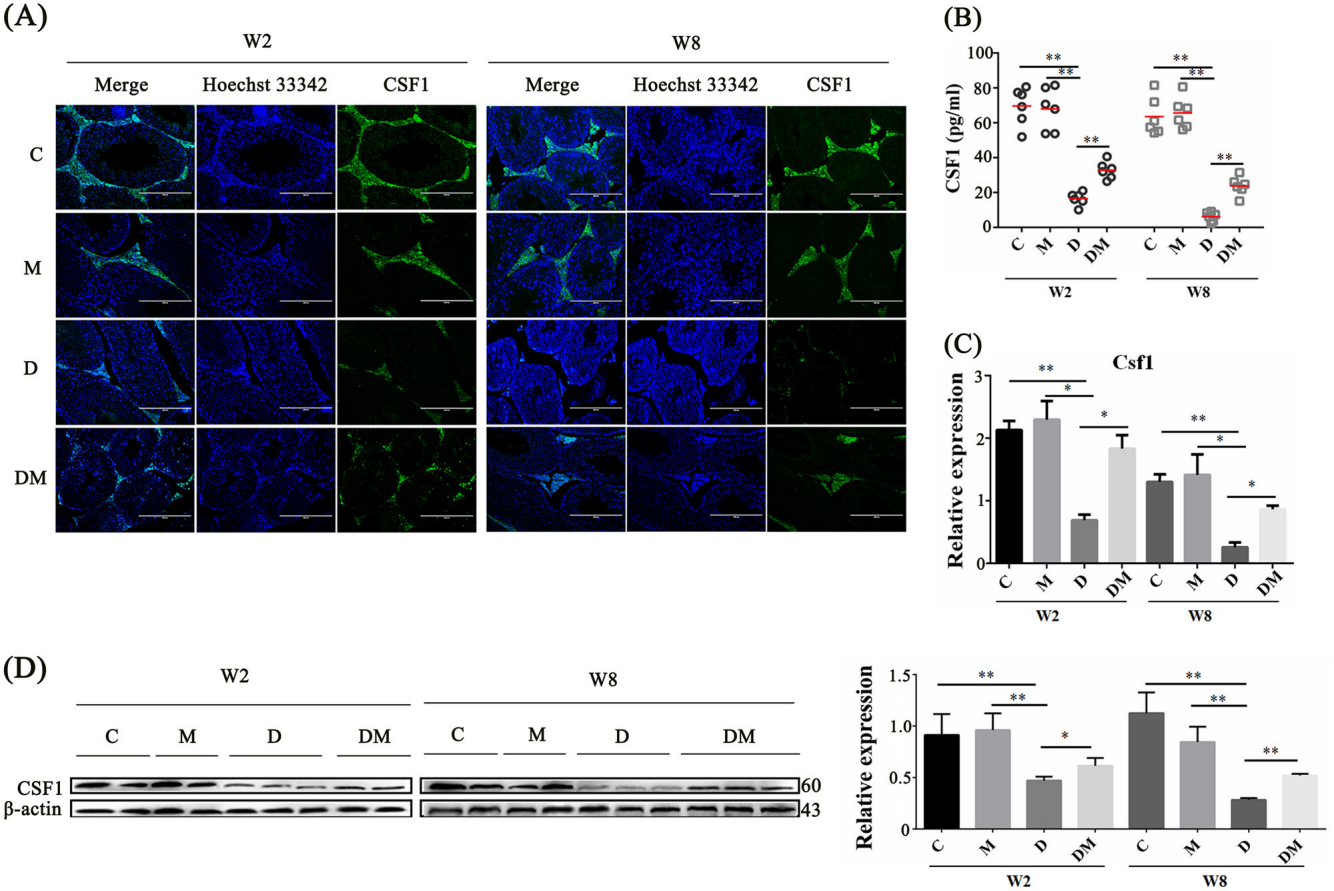
Diabetes suppressed CSF1 expression in testes recovered by melatonin. **a** CSF1 expression with Hoechst 33342 and their merges of testicular sections from mice under four different treatments in the short term (W2, 2 weeks) and long term (W8, 8 weeks) experiments. Scale bar, 200 μm. **b** ELISA of CSF1 in mice serum of the four different groups in W2 and W8 experiments. **c** RT-qPCR detected *Csf1* expression in mice testicular tissue under the four different treatments in W2 and W8 experiments. **d** CSF1 expression level detected by western blot analysis in the four groups in W2 and W8. Histograms are the statistical result of Image J (V1.48d) gradation analysis for the western blot experiments. (The results are shown as the mean ± S.E.M of at least three mice (*n* = 3) and the statistical significance was expressed as follows: *p < 0.05; **p < 0.01). C control group, M normal group treated with MLT, D diabetic group, DM MLT-treated diabetic group

The mRNA and protein expression levels of CSF1 in mouse testes were using immunofluorescent staining, RT-qPCR, and Western blot. The CSF1 protein levels in the diabetic mice were significantly lower than those in the control mice (Fig. 7a). The proportion of CSF1-positive cells increased in the DM group in short- and long-term experiments. Compared with that in the normal group, the serum CSF1 was increased significantly in the melatonin-treated group (Fig. 7b). In Fig. 7c, the mRNA expression level of CSF1 was sharply reduced in the testicular cells of the mice with diabetes in short- and long-term experiments. By comparison, the expression of these genes was elevated in the mice treated with melatonin. Consistent with the mRNA expression pattern, the CSF1 protein expression was downregulated in the testicular cells of the mice with diabetes and restored significantly in the long term melatonin treatment groups (*P* < 0.01) (Fig. 7d). These results reveal that some of Leydig cells lost their CSF1 synthesis and secretory functions in diabetes in long- or short-term experiments.

### ERS suppressed CSF1 expression in Leydig cells

**Fig. 8:**
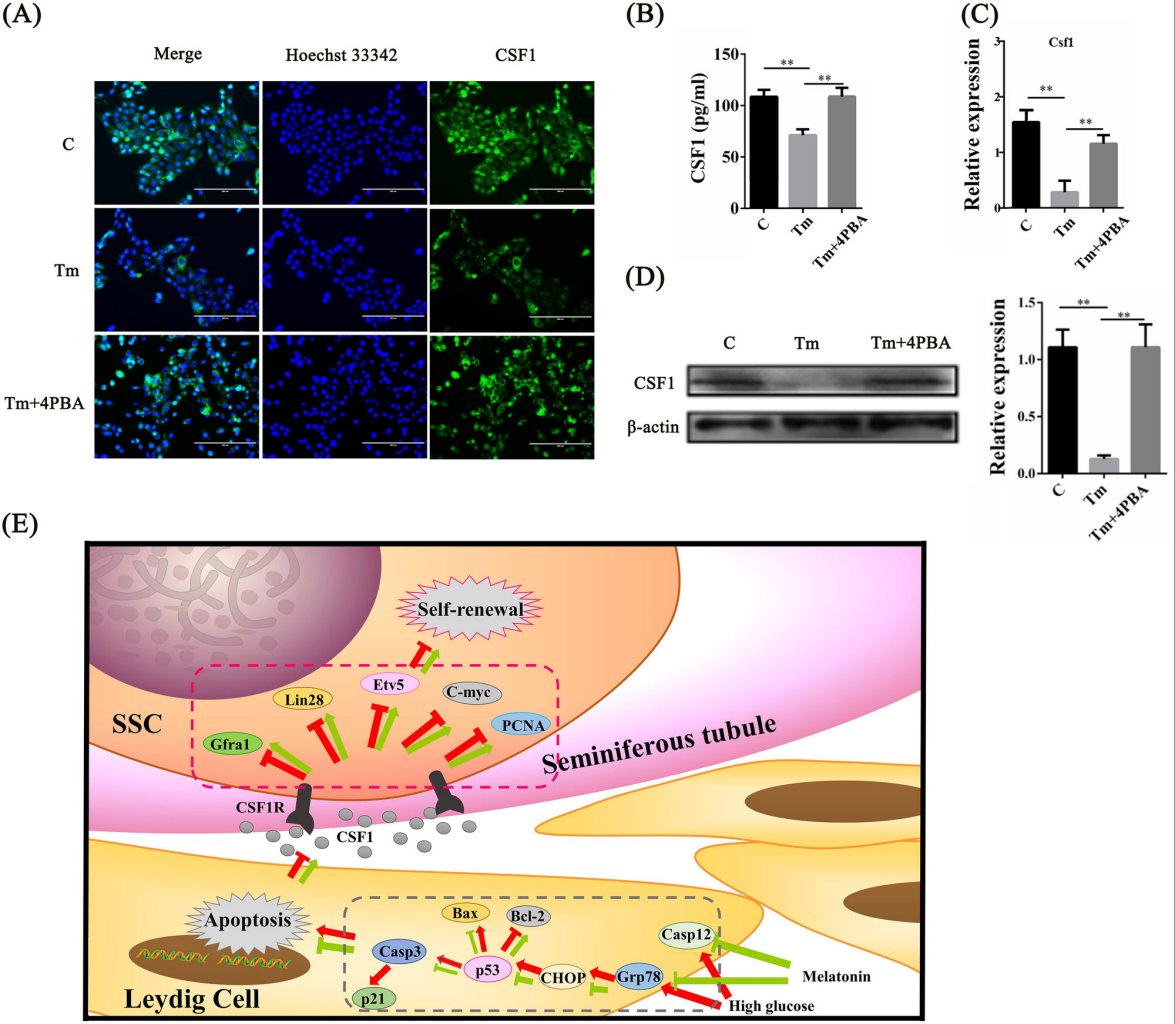
ERS suppresses the secretion of CSF1 in Leydig cell line. **a** CSF1 expression with Hoechst 33342 and their merges of MLTC-1 under three different ERS states (C control, Tm Tm treatment induced excessive ERS, Tm + 4PBA Tm treatment in combination with 4PBA for alleviated ERS). **b** CSF1 concentration in culture medium of MLTC-1 under the three different treatments was detected using ELISA. **c** RT-qPCR detected the mRNA expression level of *Csf1* in MLTC-1 under the three treatments. **d** Expression of CSF1 in MLTC-1 under different treatments was measured by Western blot. Histograms are the statistical result of Image J (V1.48d) gradation analysis for the western blot experiments. **e** A graphical conclusion of this study is displayed: DM-induced high glucose activated ERS response factors (Grp78 and CHOP) in Leydig cells, resulting in apoptosis of Leydig cells and growth arrest of SSCs in testes. In response to these changes, melatonin treatment alleviated the apoptosis of Leydig cells via inhibition of ERS and recovered the CSF1 secretion in testes. CSF1 then resumed the self-renewal capacity of SSCs. (The results are expressed as the mean ± S.E.M of three separated wells of cells (*n* = 3) in at least three different experiments and the statistical significance is expressed as follows: *p < 0.05; **p < 0.01)

To determine whether ERS downregulated CSF1, the expression of CSF1 was detected in MLTC-1 cells treated with 4PBA and Tm. In Fig. 8a, excessive Tm-induced ERS sharply decreased the proportion of CSF1-positive cells, but this parameter was improved by treating the cells with Tm in combination with 4PBA. Similar results were confirmed using ELISA (Fig. 8b). To further verify these findings, CSF1 expression was also determined through RT-qPCR and Western blot. Consistent with the pattern illustrated in Fig. 8a, b, the mRNA and protein expression levels of CSF1 were downregulated in the MLTC-1 cells exposed to excessive ERS, while restoring ERS (Tm combined with 4PBA) improved CSF1 expression (Fig. 8c, d). Moreover, the CSF1 receptor (CSF1R) was also detected. In Fig. S5, the positive rate of CSF1R in SSCs incubated on the feeder layer under ERS was not significantly different from that of the control group.

## Discussion

DM accounts for the highest incidence among chronic metabolic diseases which pose a risk to human health, and induces major damage to many systems and organs^32^. The abnormal glucose metabolism plays an important role in the molecular lesions and organ pathology. Especially, Type 2 DM (T2DM), has become increasingly prevalent among children/adolescents and adults of reproductive age and results in high incidence of male infertility which emerges as a pressing health issue^33,34^. Studies have reported a link between DM and male infertility. DM causes histopathology damage to testes^35^ and contributes to male infertility by triggering abnormal spermatogenesis, which results in impaired sperm parameters^36^.

The high blood glucose caused by DM changes glucose metabolism in the testis. Leydig cells and SSCs are destroyed under the high glucose conditions^35^. We used STZ-induced diabetic mice to examine diabetes-induced male infertility^20^, and explore the mechanism of infertility resulted from T2DM; the data demonstrated that hyperglycemia in STZ-induced diabetic mice was accompanied by the loss of Leydig cells and SSCs. Apoptosis is associated with diabetes, and ERS is one of the main causes of apoptosis. The apoptosis activated in the STZ-induced diabetic mice testis and epididymis was mediated by ERS^37^. High-glucose-induced ERS is also closely linked to various aspects of cell dysfunctions in patients with diabetes^38,39^. In Akita mice, ERS directly induces β-cell death and dysfunction^40–43^. ERS adversely affects the insulin-secreting function of β-cells in the pancreas, and the insulin deficiency consequently induces hyperglycemia^44^. One report documented that the ERS in the Drosophila male accessory gland results in infertility^45^. Hence, ERS-induced apoptosis in testis is an important consideration for infertility resulted from diabetes. In our study, we found that hyperglycemia augmented ERS and apoptosis of Leydig cells, and disturbed the homeostasis of SSCs niches in STZ-induced diabetic mice. Thus, it was likely that the ERS induced by hyperglycemia caused the loss of both the Leydig cell and SSCs^46^.

Melatonin, a compound secreted by the pineal gland, functions as an antioxidant and free radical scavenger^47–51^, and has a widespread clinical application^52,53^. In addition to oxidative stress inhibition, ERS is slightly decreased by melatonin after a prolonged high glucose treatment in pancreatic β-cells^39,54,55^. Melatonin directly and indirectly ameliorates ERS in multi-pathologies^56^. Melatonin directly mediates insulin biosynthesis in INS-1E cells under induced ERS^57^. Melatonin also functions as an inhibitor of lipopolysaccharide-mediated placental ERS in mice^58^ and significantly attenuates Cd-induced ERS in mouse testes^59^. Melatonin also has anti-inflammatory effects via ERS in acute pancreatitis^60^. Thus, melatonin may repair the adverse effects of hyperglycemia on Leydig cells and SSCs by inhibiting ERS. We found that melatonin reduced the apoptosis rate of cells in the testes by inhibiting expression of Grp78, CHOP, and Caspase12 which were related to ERS in STZ-induced diabetic mice. Moreover, the in vitro cell culture assay demonstrated that MLTC-1 was more sensitive to high glucose than SSC lines and thus they underwent apoptosis. The expression levels of ERS-related genes, including Grp78, Chop, and Caspase12, increased at high glucose levels in MLTC-1 cells. Thus, high blood glucose seems to promote ERS in Leydig cells. Subsequently, the dysfunction of Leydig cells might impact the status of SSCs. When MLTC-1 cells were treated with melatonin, both the ERS and apoptosis were alleviated in high glucose environment. Flow cytometry analysis further showed that melatonin decreased the high apoptotic rate of testicular cells. These results indicated that ERS supported by a high glucose environment induces apoptosis of Leydig cells and contributes to the progression of the injury to the male reproductive system. In combination with the inhibitor and activator of ERS, i.e., 4PBA and Tm, treated MLTC-1 when co-cultured with SSCs, the self-renewal and proliferation of SSCs were closely related to the ERS status of MLTC-1. These findings indicate that melatonin overcomes the hyperglycemia-induced ERS, while the ERS promotes apoptosis of Leydig cells in STZ-induced diabetic mice. Thus melatonin improved the male reproductive physiology.

Testes produce sperm throughout the male reproductive lifespan by balancing the self-renewal and differentiation of SSCs^61^. The regeneration and differentiation of SSCs were supported by the Leydig cells^62^. Leydig cells secrete essential hormones and cytokines to maintain male reproductive functions. Testosterone is the main resource of androgens in the testis, which is produced by Leydig cells but regulated mainly by cytokine secreted by Sertoli cells. Melatonin acts as a local modulator of the endocrine activity in Leydig cells, and melatonin is a key player in the regulation of steroidogenesis^63^. We hypothesize that MLT improved the cytokines secretion processes that were initially destroyed by ERS in Leydig cells, and then repaired the function of SSCs. Cytokines, such as CSF1, are expressed by macrophages on the surface of seminiferous tubules, near the SSC niche enriched by undifferentiated spermatogonia^61^. CSF1 is an extrinsic stimulator of SSC self-renewal and Leydig cells are contributors to the testicular stem cell niche in mammals^15^. In the current study, the inhibition of CSF1 expression and secretion caused by ERS in testes and MLTC-1 were both improved by melatonin. However, no changes of CSF1R level in SSCs were noted. Thus, it was inferred that melatonin increased the CSF1 secretion of Leydig cells and promoted the self-renewal of SSCs. CHOP-induced apoptosis in ERS has been implicated in diabetes^64^; we also found that high glucose treatment upregulated the expression of CHOP and Grp78 and activated apoptosis in Leydig cells. The loss of Leydig cells resulted in a deficiency of CSF1 secretion in the SSC niche, leading to the SSCs self-renewal capacity damage. This study is the first to demonstrate that CSF1 is implicated in the role of melatonin maintaining SSC self-renewal under high glucose conditions.

In conclusion, melatonin reduced Leydig cell apoptosis induced by ERS and increased the secretion of the cytokine, CSF1, which improved the reduced spermatogenesis caused by diabetes. Our studies suggest a novel therapy for hyperglycemia-induced male infertility from the standpoint of the source of mature sperm, which are promoted by melatonin therapy. Further study is needed to determine whether the SSC self-renewal capacity is directly or indirectly promoted by the melatonin therapy.

## Experimental procedures

### Animals and animal experimental protocol

Figure 1 provides the schematic design of the experimental protocols. The five-week-old ICR male mice were divided into four groups. Group D mice served as STZ diabetic group. They were fed with normal mouse chow and received a single intraperitoneal injection of STZ diluted in citric acid buffer (100 mg/kg body weight) on day 1. The effects on sperm cell developmental physiological and biochemical properties were tested after 2 or 8 weeks of STZ injection. Group M mice served as melatonin-treated group. They were fed with normal mouse chow and treated with a daily single dose of melatonin (10 mg/kg/d) diluted in saline through gavage^65–67^. The disposal time points are shown in Fig. 1. Group DM mice served as STZ-MLT-treated group. They were fed normal chow and injected intraperitoneally with single dose of STZ as in group D mice and administered also with a daily single dose of melatonin 10 mg/kg through gavage. Group C mice (control group) were fed with normal mouse chow and given a daily single dose of saline as the solvent administered to the MLT-treated group via gavage. Each group consisted of 4–8 mice.

### Isolation of germ cells and the experimental protocol

Mouse SSCs were isolated from testes at Day 6 postnatal mice. The tunica albuginea was separated under the stereomicroscope and testis was digested with collagenase IV (Invitrogen, Carlsbad, CA, USA) for 15 min and pipetted repeatedly for 5 min and then centrifuged at 1200 rpm. The seminiferous tubules were isolated from mouse testes using a small surgical scissors. The male germ cells were separated using a second enzymatic digestion with 4 mg/ml collagenase IV (Invitrogen), 2 mg/ml trypsin (Invitrogen) and 1 µg/ml DNase I (Invitrogen). The cell suspension was incubated for 1 h at 37 °C in gelatin-coated dish, 1 h at 37 °C and then in laminin-coated dish to eliminate residual adherent Sertoli cells (differential plating). Then the SSCs were collected by centrifugation at 1400 rpm for 5 min. The primary cells were seeded as 1 × 10^5^ cells/ml onto mitotically inactivated mouse embryonic fibroblast (MEF) cell feeders (5 × 10^4^ cells/cm^2^). Then the cells were maintained at 37 °C in a humidified 5% CO_2_, 95% air atmosphere. After 1–2 weeks, large multicellular (>100 cells) colonies that formed on the dish were sequentially passaged every 3 days. The medium was changed every two days and cells were subcultured using TrypLE (Invitrogen) at a 1:2–3 dilution. SSCs were cultured in α-MEM medium, supplemented with 10% FBS (Hyclone, Logan, UT, USA), 0.1 mM 2-mercaptoethanol (Invitrogen), 4 mM glutamine (Invitrogen), 1% non-essential amino acids (Invitrogen), 10 ng/ml GDNF (Peprotech, Rocky Hill, NJ, USA), 20 ng/ml EGF (Millipore, Temecula, CA, USA), 20 ng/ml bFGF (Millipore) and 1000 U/ml LIF (Millipore) and incubated at 37 °C in 5% CO_2_ balance air atmosphere.

C18-4 cells (from Mus musculus, a spermatogonial stem cell line) were cultured with Dulbecco’s Modified Eagle’s Medium/Nutrient Mixture F12 (DMEM/F12, Gibco, Grand Island, NY, USA) supplemented with 10% FBS (Hyclone), 2 mM L-glutamine (Invitrogen), and 100 U/ml penicillin and streptomycin (Invitrogen)^68^. GC1 (Mus musculus, BALB/c, established, ATCC number: CRL-2053^TM^) cells were cultured with Dulbecco’s Modified Eagle’s Medium (DMEM, Gibco) supplemented with 10% FBS (Gibco), 2 mM L-glutamine (Invitrogen), and 100 U/ml penicillin and streptomycin (Invitrogen)^69^. MLTC-1 cells (Mus musculus, C57BL/6, established cell line, ATCC number: CRL-2065^TM^) were cultured with 1640 Medium (RPMI-1640, Gibco) supplemented with 10% FBS (Gibco), 2 mM L-glutamine (Invitrogen), and 100 U/ml penicillin and streptomycin (Invitrogen). The cells were passaged every 3–4 days and maintained at 37 °C in a humidified 5% CO_2_ incubator. The cells were dissociated by 0.25% trypsin-EDTA (Invitrogen) and reseeded into multi-well plates for the subsequent experiment, the medium was changed every 2 days. The cells were cultured 24 h prior to experiments at the cell density of 5 × 10^4^ cells/cm^2^ approximately.

Melatonin was dissolved in DMSO physiological saline to 10^−3^ M. Tunicamycin (Tm) (Sigma, St. Louis, MO, USA) and 4-phenyl butyric acid (4PBA) (Sigma) were dissolved in DMSO and prepared at 10 μM and 1 M concentrations, respectively.

### Immunofluorescence staining

The cells cultured in 48-well plate were fixed with 4% formaldehyde for 10 min at room temperature (RT) and were washed with PBS × 3, 5 min each time. The cells were permeabilized by 0.1% Triton X-100 for 10 min at RT and were blocked for minimum 30 min with 1% BSA at RT. Then the cells were incubated with primary antibodies specific against PCNA (1:100, mouse IgG, Sangon, Shanghai, PRC), PLZF (1:300, mouse IgG, Abcam, Cambridge, UK), CSF1 (1:100, rabbit IgG, Sangon), CSF1r (1:100, mouse IgG, Sangon), respectively, for overnight at 4 °C. After three washes with PBS, the cells were incubated with the corresponding secondary antibodies (1:500, Millipore Chemicon, Temecula, CA, USA) at RT for 1 h, followed by three washes in the same buffer. They were then incubated with Hoechst 33342 (Sigma) at RT for 2 min. Concurrently, the negative controls were stained with conjugated secondary antibodies alone. Images were captured with Evos f1 fluorescence microscope (AMG, Millcreek, Washington, USA).

Tissues were fixed in 4% (w/v) paraformaldehyde in 0.01 M PBS (pH 7.4), washed in PBS, dehydrated in ethanol (70%, 90%, and 100%) and embedded in paraffin wax. Testicular and epididymal sections (5 µm) were rehydrated (xylene 5 min; ethanol 100%, 95%, 70%, 5 min each) and washed in distilled water prior to immunofluorescence staining.

### BrdU incorporation assay

The proliferative ability of MLTC-1 and SSCs was evaluated by BrdU incorporation assay. Briefly, cells were treated with 30 μg/ml BrdU (Sigma, St Louis, MO, USA) for 6 h before BrdU immunostaining. Then cells were fixed in 4% paraformaldehyde (PFA) for 15 min at RT and washed three times (5 min/each time) with PBS (pH 7.4) containing 0.1% Triton X-100. The cells were then washed for three times with PBS (pH 7.4) alone. Anti-BrdU antibody (1:100; Santa Cruz, CA, USA) dissolved in 0.1 M PBS (pH 7.4) containing 5% goat serum was added. After being incubated overnight at 4 °C cells were washed in PBS (pH 7.4) three times and then incubated with the corresponding secondary antibody (1:500, FITC, Millipore) for 1 h at room temperature. After three washes with PBS, cells were visualized under an AMG fluorescent microscope and analyzed for BrdU uptake. Under the microscope magnifying 100, each sample in four random field sights were selected to conduct the quantification of the image analyses. The percentage of BrdU-positive cells was calculated as the number of BrdU-positive cells out of the total number of cells (×100).

### CCK-8 assay

For CCK-8 assay (Vazyme Biotech Co., Ltd, Nanjing PRC), MLTC-1 cells were seeded on 96-well plate. Cells were incubated in CCK-8 solution for 1 h at 37 °C. The amount of formazan dye was measured by examining the absorbance at 450 nm with a microplate reader.

### RT-qPCR

Quantitative reverse transcriptase-polymerase chain reaction (RT-qPCR) was set up in 25 μl reaction mixtures containing 12.5 μl 1 x SYBR Premix ExTaq^TM^ (Bioer, Hangzhou, PRC), 0.5 μl sense primer, 0.5 μl antisense primer, 11 μl distilled water, and 0.5 μl template. Reaction conditions were as follows: 95 °C for 30 s, followed by 45 cycles at 95 °C for 5 s, and 59 °C for 20 s. The expression levels of mRNAs were normalized to Gapdh in each well.

### TdT-mediated dUTP-X nicked end labeling (TUNEL assay)

Cells were incubated for 15 min with proteinase K (20 μg/ml, Roche, Basel, Switzerland) at RT. Then cells were washed three times with PBS and incubated for 60 min at 37 °C in a moist chamber with 50 μl TUNEL mix (5 μl Terminal Deoxynucleotidyl Transferase (TdT) and 45 μl fluorescein-dUTP), negative control was just added 50 μl fluorescein-dUTP. After washing for three times with PBS for 5 min each at RT, the cells were incubated with Hoechst 33342 for 2 min at RT. After three PBS washes, cells were visualized under an AMG fluorescent microscope. The percentage of TUNEL-positive cells was calculated as (the number of TUNEL-positive cells out of the total number of cells) × 100.

### Western blot analysis

The tissue or cell samples were collected and lysed with lysis buffer, and then protein extractions were obtained. The protein was quantified by using a BCA Protein Assay kit (Beyotime, Shanghai, PRC). After denaturation by heating for 10 min at 100 °C in 5% SDS-PAGE loading buffer, the protein sample was resolved by SDS-PAGE and transferred to a PVDF membrane (61). The primary antibodies used for Western blot analysis included anti-P53 antibody (1:1000, mouse IgG, Sino Biological Inc., Beijing, PRC), anti-Bcl2 antibody (1:1000, Sino Biological Inc.), anti-Bax antibody (1:1,000, rabbit IgG, Cell Signaling Technology, Danvers, MA, USA), anti-Caspase 3 antibody (1:1000, mouse IgG, Cell Signaling Technology), anti-Chop antibody (1:1,000, mouse IgG, Sangon), anti-Grp78 antibody (1:1000, rabbit IgG Sangon), anti-CSF1 antibody (1:1000, rabbit IgG, Sangon), anti-CSF1r antibody (1:1000, mouse IgG, Sangon), and anti-β-actin antibody (1:5,000, mouse IgG2b, Cell Signaling Technology). The protein bands were detected using a Bio-Rad imaging system (Bio-Rad, Hercules, CA, USA) and quantified using Image J (V1.48d).

### Cell cycle and apoptosis assay by flow cytometry

Cell cycle distribution was assessed by flow cytometry after PI staining. Adherent cells were dissociated by 0.25% trypsinization and detached cells were fixed in 70% ethanol at 4 °C for 24 h. After being washed with PBS, samples were stained with 50 μg/ml PI and 50 μg/ml RNase A at 37 °C for 20 min, then analyzed by flow cytometry (Beckman Altra, Beckman Coulter Inc., Brea, CA, USA). At least 5000 cells were collected per sample.

Cell apoptosis distribution was assessed after Annexin V-FITC and PI staining. Cells were harvested and washed in cold PBS and cold binding buffer x1. They were then re-suspended in cold 1 x binding buffer, 1 × 10^6^ cells/ml. 100 μl cell suspension (1 × 10^5^ cells) were added to each labeled tube, 5 μl Annexin V-FITC was added to appropriate tubes. Each tube was gently vortexed and incubated for 10 min at room temperature. 5 ml PI solution was added then and those tubes were stabilized for 5 min at room temperature, protected from the light. Before the analysis, cells were washed once in PBS and resuspended with PBS. Flow cytometer was utilized to perform the measurements experiment (Beckman Altra). The fluorescence intensity was also observed under fluorescence microscope.

### Enzyme linked immunosorbent assay (ELISA)

After snipping the tail tip, the blood sample was collected and plasma glucose levels were determined using the blood glucosemeter (Yicheng, Beijing, China). Animals were fasted for 12 h before the measurement. Mouse CSF1 level of mouse plasma was quantified using an ELISA kit (Boster, Wuhan, China) according to the instructions.

### Statistical analysis

One-way analysis of variance (one-way ANOVA) was used and post-tests were conducted using Newman–Keuls multiple range test, if *P*-values are significant. Students’ *t*-test was used when only two pairs of data were compared. All data are presented as mean ± SD, and statistical significance was expressed as follows: *P < 0.05; **P < 0.01. All data are representative of at least three different experiments and were analyzed using Graphpad Prism software (La Jolla, CA, USA).

## Electronic supplementary material

Figure S1

Figure S2

Figure S3

Figure S4

Figure S5

supplementary figure legends
