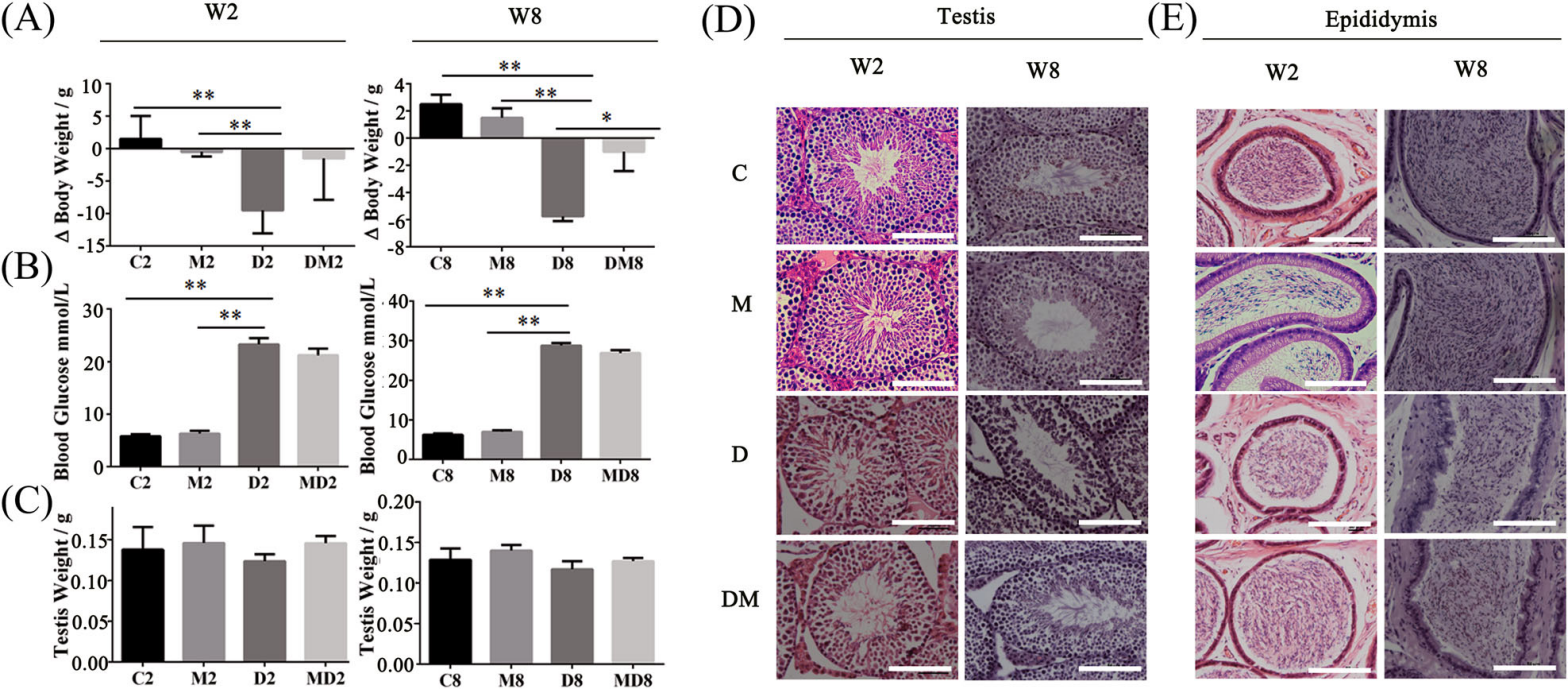# Correction to: Melatonin attenuates detrimental effects of diabetes on the niche of mouse spermatogonial stem cells by maintaining Leydig cells

**DOI:** 10.1038/s41419-024-07161-z

**Published:** 2024-10-28

**Authors:** Zhaoyu Du, Shuanshuan Xu, Shuxian Hu, Hong Yang, Zhe Zhou, Kuldip Sidhu, Yiliang Miao, Zhonghua Liu, Wei Shen, Russel J. Reiter, Jinlian Hua, Sha Peng

**Affiliations:** 1 College of Veterinary Medicine, Shaanxi Centre of Stem Cells Engineering & Technology, Northwest A&F University, Yangling, 712100 Shaanxi China; 2 Centre for Healthy Brain Ageing, UNSW Medicine, Randwick, NSW 2031 Australia; 3 College of Animal Science & Technology, College of Veterinary Medicine, Huazhong Agricultural University, 430070 Wuhan, China; 4 College of Life Science, Northeast Agricultural University, 150036 Harbin, China; 5 College of life sciences, Institute of Reproductive Sciences, Qingdao Agriculture University, 266109 Qingdao, China; 6 Department of Cell Systems and Anatomy, UT Health, San Antonio, TX 78229-3900 USA

Correction to: *Cell Death and Disease* 10.1038/s41419-018-0956-4, published online 20 September 2018

Upon investigation, it has come to our attention that there was a mix-up in the figures labeled Fig. 2 (D) and (E) between the Control group (C) and the Melatonin group (M) from the 2-week samples (both testis and epididymis H&E). We found that this issue was caused by a disorganized scanning process of the tissue slides, and we carelessly ignored the mistake because these are in the health negative controls which were not expected to be significantly different in the context of pathological affects. To address this, the pathological tissue blocks have been retrieved from the sample repository and rescanned, and the data has been subsequently verified.

Correct figure