# Supplementary figures and images for: Melatonin attenuates detrimental effects of diabetes on the niche of mouse spermatogonial stem cells by maintaining Leydig cells
Source: Cell Death Dis. 2018 Sep 20;9(10):968. doi: 10.1038/s41419-018-0956-4 (PMC6148071; doi:10.1038/s41419-018-0956-4)

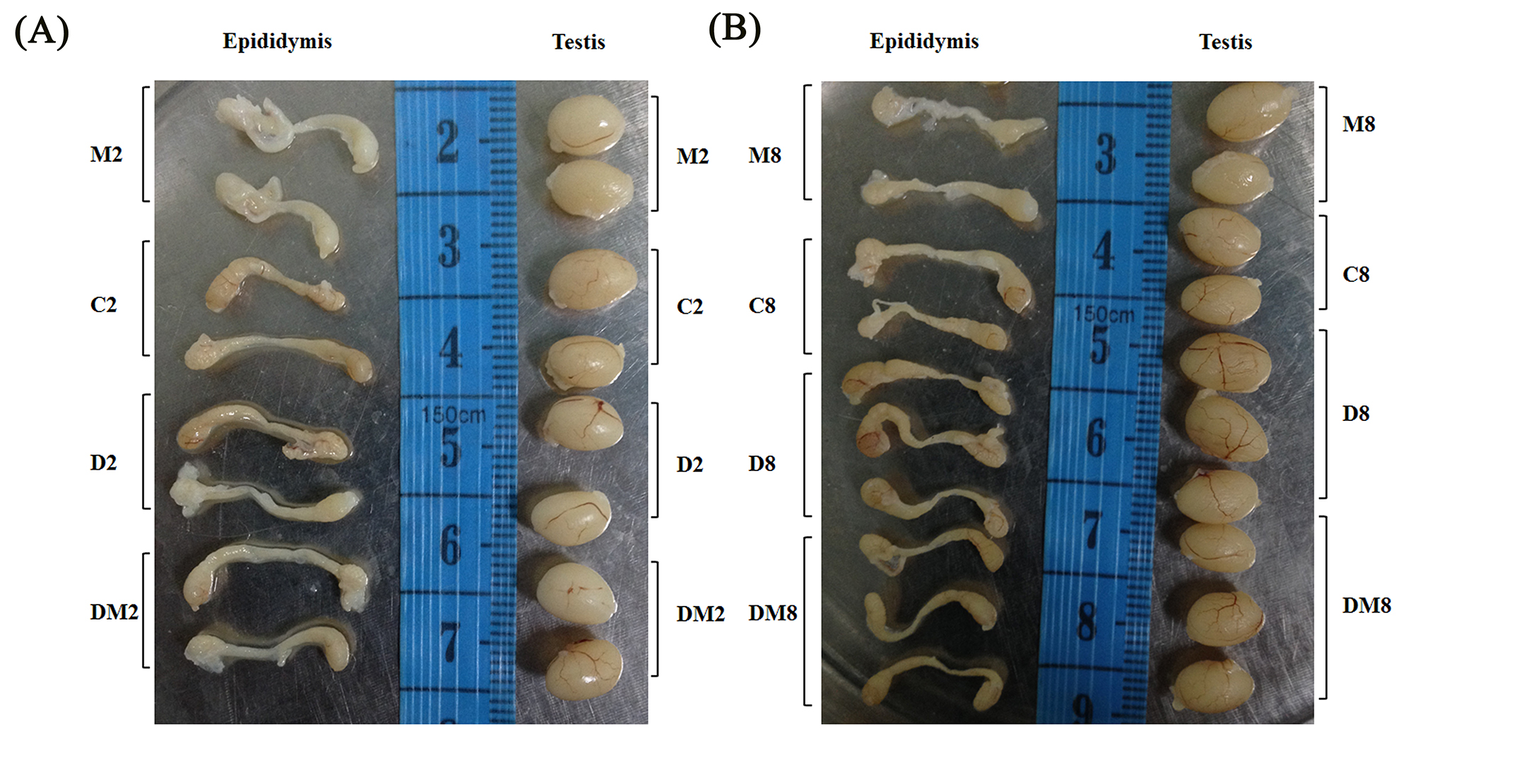

Supplement: Supplementary file 1 — Figure S1 [file 41419_2018_956_MOESM1_ESM.jpg]

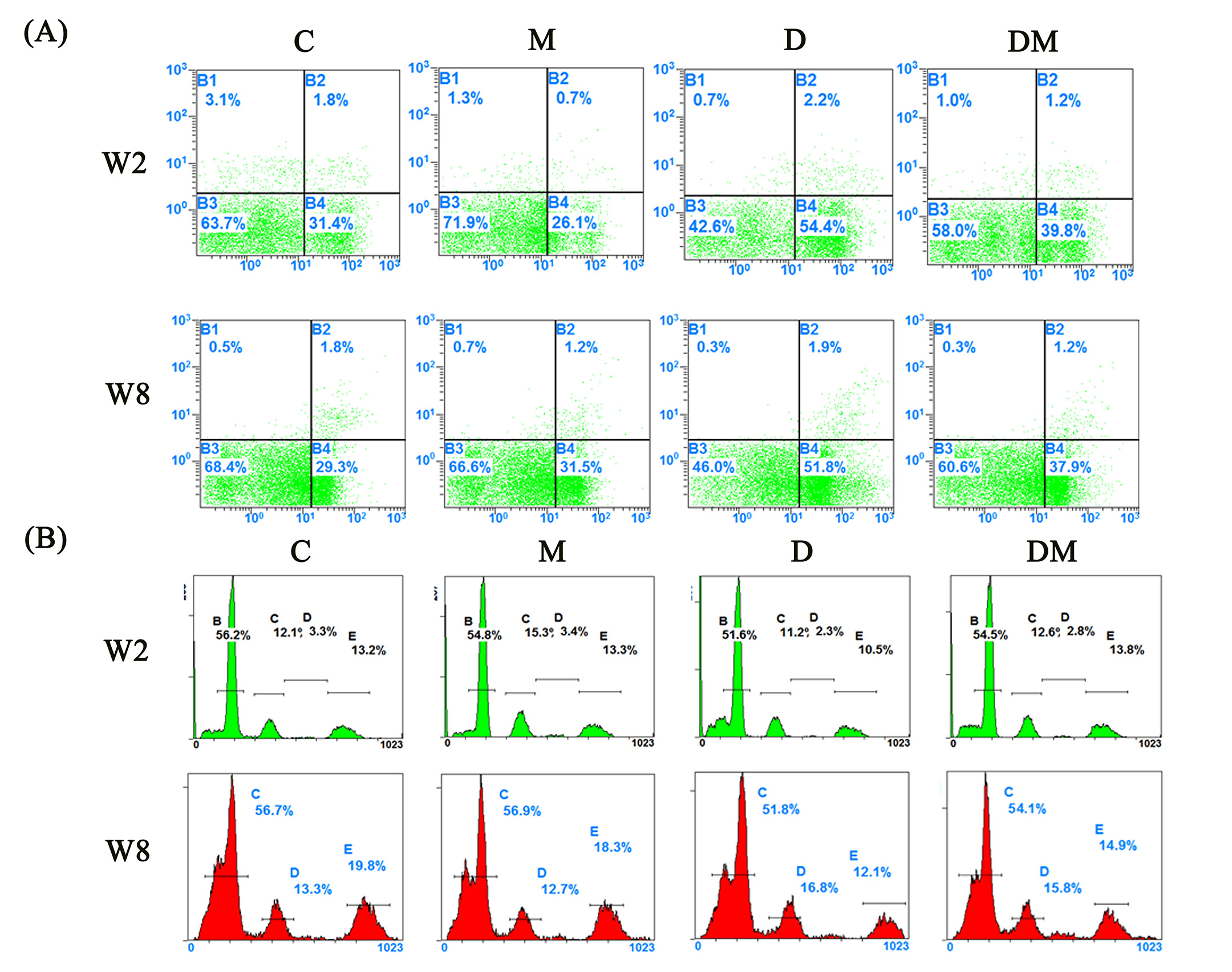

Supplement: Supplementary file 2 — Figure S2 [file 41419_2018_956_MOESM2_ESM.jpg]

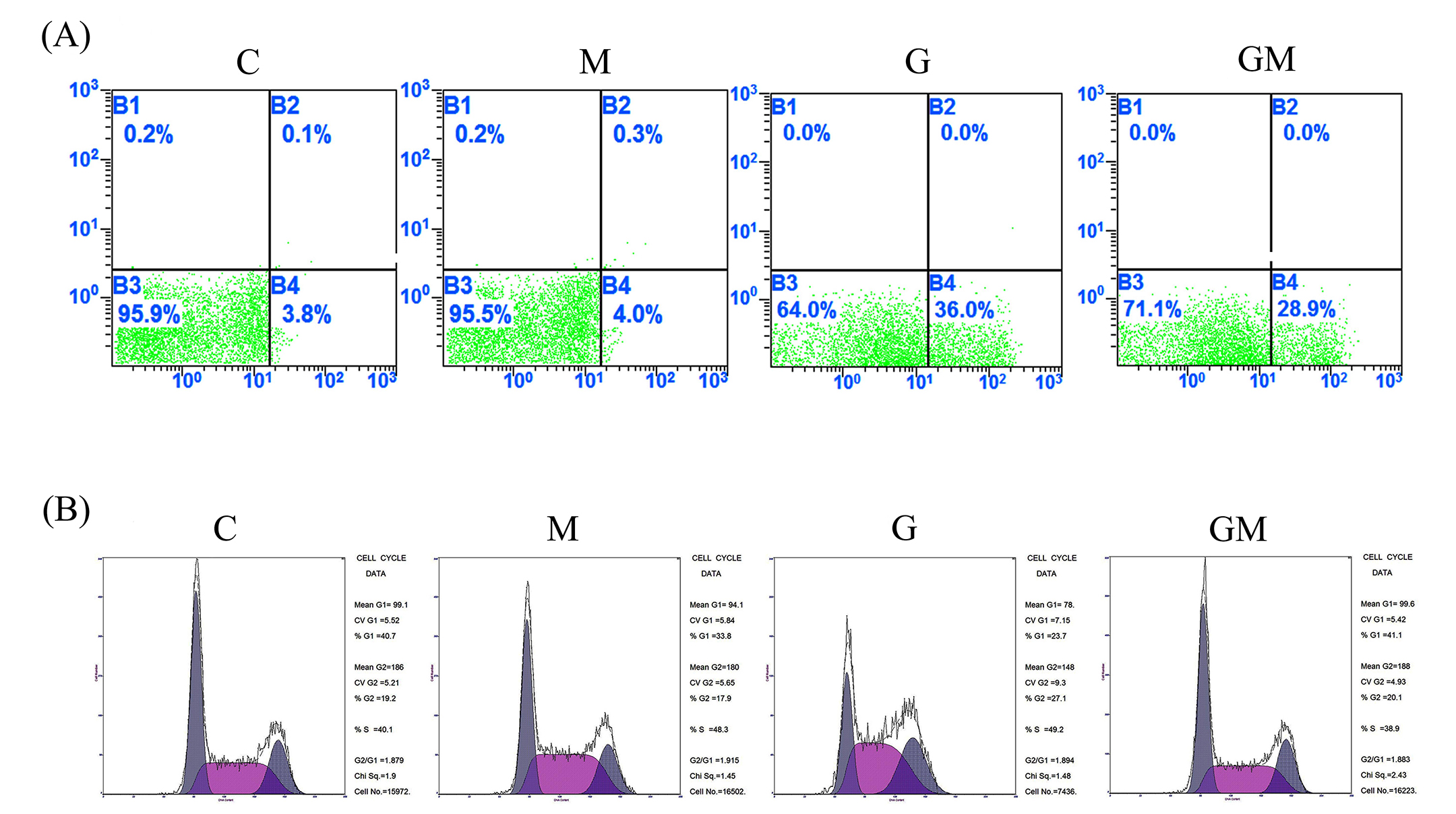

Supplement: Supplementary file 3 — Figure S3 [file 41419_2018_956_MOESM3_ESM.jpg]

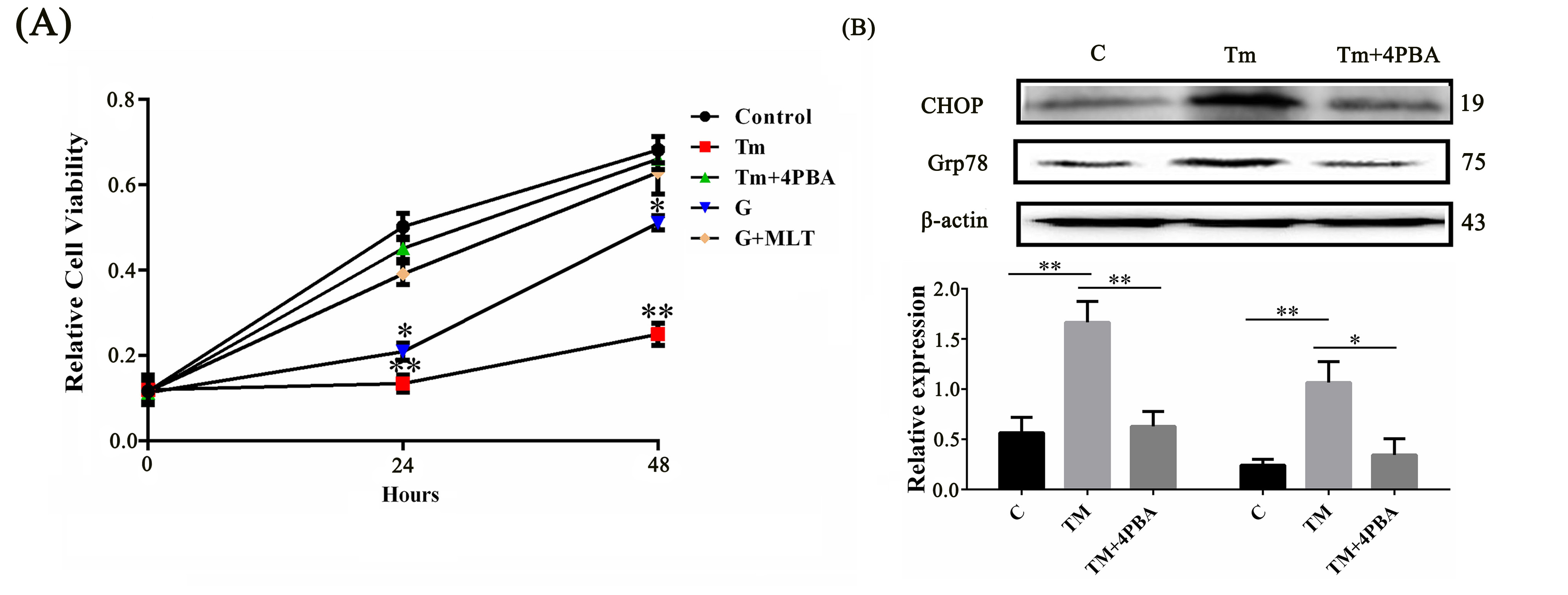

Supplement: Supplementary file 4 — Figure S4 [file 41419_2018_956_MOESM4_ESM.jpg]

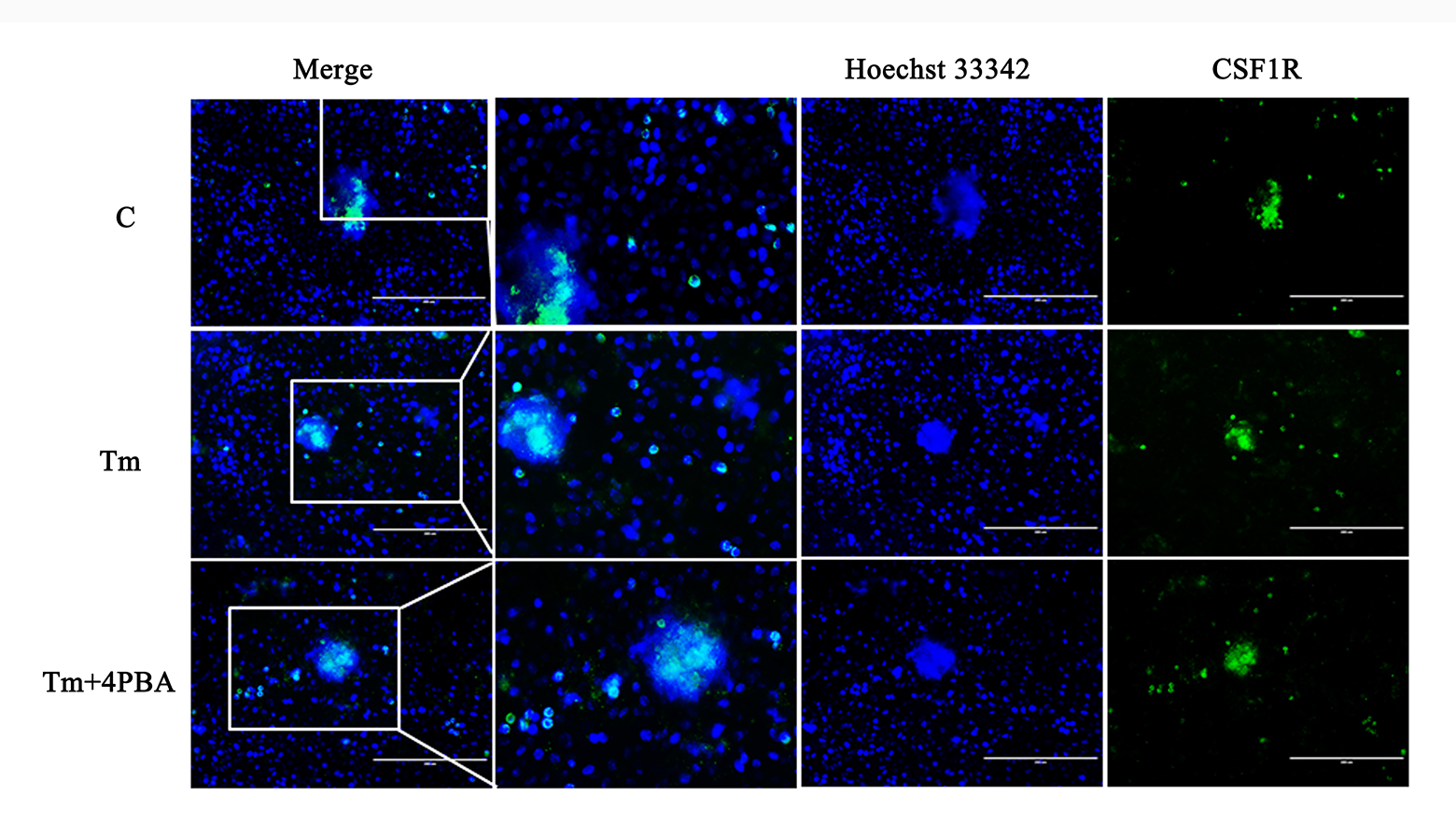

Supplement: Supplementary file 5 — Figure S5 [file 41419_2018_956_MOESM5_ESM.jpg]
