# Supplementary material for: Melatonin attenuates detrimental effects of diabetes on the niche of mouse spermatogonial stem cells by maintaining Leydig cells
Source: Cell Death Dis. 2018 Sep 20;9(10):968. doi: 10.1038/s41419-018-0956-4 (PMC6148071; doi:10.1038/s41419-018-0956-4)
Supplement: Supplementary file 6 — supplementary figure legends [file 41419_2018_956_MOESM6_ESM.docx]

**Figure. S1** Shape and size of testis and epididymis after short term （W2, 2 weeks）or long term (W8, 8 weeks) treatments.

**Figure. S2** (A) Testicular cell apoptosis rate and (B) nuclear DNA ploidy in short term (W2, 2 weeks) and long term experiments (W8, 8 weeks) were analyzed by flow cytometry.

**Figure. S3** The effects of MLT on (A) apoptotic rate and (B) the cell cycle distribution were detected by flow cytometry (n = 3).

**Figure. S4** (A) Tunicamycin (Tm) was used to activate excessive ERS in MLTC-1 cell line, while 4-phenylbutyric acid (4PBA) was used as an inhibitor. CCK8 assay illustrated the differences of relative cell viability treated with Tm, 4PBA, high glucose and melatonin for 0 h, 24 h and 48 h. (B) Western blot analysis revealed the expression of ERS-related genes. Histograms are the statistical result of Image J (V1.48d) gradation analysis for the western blot experiments. (The results are expressed as the mean ± S.E.M of three separated wells of cells (n = 3) in at least three different experiments and the statistical significance is expressed as follows: *p< 0.05; **p < 0.01).

**Figure. S5** CSF1R expression with Hoechst 33342 and their merges in SSCs growing on the feeders of three different ERS states. Scale bar, 200 μm.
